# Nanoparticle Polymeric Micellar Paclitaxel Versus Paclitaxel for Patients with Advanced Gastric Cancer

**DOI:** 10.1007/s12029-024-01058-y

**Published:** 2024-04-26

**Authors:** Fei-Yu Wang, Xiang-Ming Huang, Yu-Qing Cao, Jie Cao, Jie Ni, Ke Li, Min Lu, Xin-En Huang

**Affiliations:** 1 Department of Medical Oncology, The Affiliated Cancer Hospital of Nanjing Medical University& Jiangsu Cancer Hospital & Jiangsu Institute of Cancer Research, No. 42 Baiziting , Jiangsu, 210009 China; 2 Affiliated Hospital of Integrated Traditional Chinese and Western Medicine, Nanjing University of Chinese Medicine, Nanjing, Jiangsu China

**Keywords:** Chemotherapy, Gastric cancer, Nanoparticle polymeric micellar paclitaxel

## Abstract

**Background:**

Nanoparticle polymeric micellar paclitaxel (NPMP) is a novel Cremophor EL (CrEL)-free nanoparticle micellar formulation of paclitaxel. This study evaluated the efficacy and toxicity of NPMP in the treatment of patients with advanced gastric cancer (AGC).

**Methods:**

Patients with histologically confirmed AGC in Jiangsu Cancer Hospital were retrospectively collected and divided into two groups. Patients in group A received NPMP at a total dose of 360 mg/m^2^ each cycle, and patients in group B were given paclitaxel at a dose of 210 mg/m^2^ each cycle. In addition, all patients received 5-fluorouracil at a dose of 0.75 g/m^2^ on days 1–4 and leucovorin at a dose of 200 mg/m^2^ on days 1–4 for at least 2 cycles.

**Results:**

From January 2021 to May 2023, 63 patients (32 in group A and 31 in group B) could be evaluated for treatment response. A marked disparity in the overall response was observed between groups A and B, indicating statistical significance*.* The overall response rate was 31% in group A (10/32) and 10% in group B (3/31) (*P* = 0.034). Disease control rate was 91% in group A (29/32) and 81% in group B (25/31) (*P* = 0.440). No statistically significant difference in adverse reactions was observed between the two groups. However, the incidence of anemia, leucopenia, nausea, vomiting, diarrhea, liver dysfunction, and allergy in group A was notably lower than that in group B.

**Conclusions:**

NPMP combined chemotherapy offers a new, active, and safe treatment for patients with AGC.

## Introduction

Gastric cancer is prevalent worldwide, with an annual occurrence exceeding one million new cases [1]. Gastric cancer ranks fourth in the incidence of malignant tumors globally and is also a major cause of malignancy-related deaths [2]. Surgery is an optimal treatment for patients with early gastric cancer [3]. However, numerous patients receive an initial diagnosis of advanced or metastatic gastric cancer. Without treatment, these patients have a poor prognosis and poor overall survival. Clinical studies suggested that systemic chemotherapy has the potential to prolong survival and enhance quality of life, in contrast to the provision of optimal supportive care [4]. Therefore, systemic chemotherapy continues to serve as the cornerstone of treatment for patients afflicted with advanced gastric cancer. Currently, regimens based on a combination of two cytotoxic agents are recommended as first-line treatment and are reported to be associated with longer patient survival compared to single-agent 5-FU [5]. National Comprehensive Cancer Network (NCCN) recommended combination regimens which include FP/XP and FOLFOX/XELOX, etc. [6]. However, there are still uncertainties regarding the choice of regimen due to individualized differences.

Paclitaxel is widely used in the treatment of gastric cancer [7]. It is the first alkaloid microtubule-targeting agent described in the literature, and its main mechanism involves disrupting microtubule dynamics, thereby inducing mitotic arrest and cell death [8]. However, paclitaxel is often formulated with Cremophor EL (CrEL) to enhance its solubility [9]. It was reported that CrEL could cause toxic effects, e.g., life-threatening allergic reactions and neuropathy [10]. Therefore, additional medications including steroids and histamine H2 receptor blockers are needed to minimize these side effects before using paclitaxel [11]. However, these medications also affect the efficacy of paclitaxel. To avoid disadvantages resulting from CrEL, new CrEL-free formulations of paclitaxel are of great interest in the clinical setting. An increasing number of researchers are seeking to alleviate this problem by synthesizing more soluble derivatives or preparing more soluble carrier-bound formulations of paclitaxel. In 2007, a CrEL-free polymeric micellar formulation of paclitaxel was introduced in clinical practice [12]. It was shown that polymeric micellar paclitaxel exhibited higher antitumor efficacy and better safety in preclinical studies compared to paclitaxel [13]. Latterly, a novel CrEL-free, nanoparticle polymeric micellar paclitaxel (NPMP) was developed in Shanghai, China [14]. And a multicenter phase III clinical trial comparing the efficacy of NPMP and paclitaxel as a first-line treatment for patients with advanced non-small cell lung cancer suggested that NPMP in combination with cisplatin achieved better survival benefits as well as a good safety profile and could be an option for patients with advanced non-small cell lung cancer [15]. However, it is unclear whether the efficacy of NPMP is superior to paclitaxel for patients with advanced gastric cancer. Hence, we undertook this retrospective study to investigate the efficacy and safety of NPMP in comparison to paclitaxel as systemic chemotherapy for advanced gastric cancer (AGC). The primary objective of this study was to assess toxic effects. The secondary objective was to assess objective tumor remission in the treatment of AGC.

## Materials and Methods

### Patient

The eligibility criteria were as follows: (I) initially pathologically diagnosed with AGC that was locally advanced (unresectable), primary metastasis, or recurrent disease; (II) measurable disease on imaging; (III) age range 18–80 years; (IV) life expectancy > 3 months; (V) normal hematologic, renal, and hepatic function; (VI) signed informed consent before chemotherapy. Patients were excluded if (I) patients failed to complete two cycles of chemotherapy; (II) patients who lacked information on post-treatment efficacy evaluation; (III) patients combined with severe consciousness disorders, severe cardiac/renal/other organ insufficiencies, blood diseases, and autoimmune system diseases; (IV) severe trauma or inflammatory infections during the treatment period; (V) pregnant or lactating women. This research was approved by the Medical Ethical Committee of Jiangsu Cancer Hospital (Jiangsu, China). The study was conducted in accordance with the Declaration of Helsinki.

### Treatment Method

Eligible patients were divided into the NPMP group (group A) and the paclitaxel group (group B). In group A, patients were treated with NPMP (Shanghai Yizhong Biotechnical Co., Ltd.), 360 mg/m^2^ by intravenous infusion (iv) each cycle. Nanoparticle polymeric micellar paclitaxel (NPMP) is a novel Cremophor EL (CrEL)-free, paclitaxel with excellent clinical performance. And NPMP (about 20 nm) can penetrate tumor cells easily and fast. In group B, patients were treated with paclitaxel, 210 mg/m^2^ each cycle. In both groups, 5-fluorouracil (Shanxi Pude Pharmaceutical Co., Ltd.) 0.75 g/m2, iv, d1-4 and calcium folinate (Shanxi Pude Pharmaceutical Co., Ltd) 200 mg/m^2^, iv, d 1–4 were included. Dexamethasone, promethazine hydrochloride, and cimetidine were used before paclitaxel administration in group B to prevent severe allergic reactions, no premedication before NPMP in group A. Dose adjustments were based on hematologic parameters and degree of non-hematologic toxicities. Chemotherapy was delayed until recovery if neutrophils fell to < 1.5 × 10^9^, or platelets fell to < 75 × 10^9^, or for patients with significant ongoing non-hematologic toxicities.

### Response Evaluation

Data from laboratory tests were collected at least once before and after starting the treatment cycle, and efficacy was assessed by radiography after two cycles of chemotherapy. CT/MRI scans were performed every two cycles. According to the World Health Organization’s criteria for identifying performance and classifying acute and sub-acute toxicities of anticancer drugs, adverse reactions were classified as grades 0–IV. In accordance with the Response Evaluation Criteria in Solid Tumors (RECIST), the response to chemotherapy was classified as complete response (CR), partial response (PR), stable disease (SD), and progressive disease (PD) [16]. The objective response rate (ORR) was defined as (CR + PR)/(CR + PR + SD + PD), while the disease control rate (DCR) was defined as (CR + PR + SD)/(CR + PR + SD + PD).

### Statistical Analysis

Descriptive statistics were used to describe treatment outcome and incidence of toxicity. SPSS27.0 statistical software was used for statistical analysis. Continuous variables such as age are evaluated with the Mann–Whitney *U* test, and Fisher’s exact test is used instead of the chi-square test when statistical power is insufficient. Statistically significant difference was set at *P* < 0.05.

## Results

From January 2021 to May 2023, 63 patients met the study criteria and were enrolled into study groups. General characteristics of patients are shown in Table 1.

**Table 1:** Patients baseline characteristics between two groups

Parameter	Polymeric micellar paclitaxel *n* = 32 (100%)	Paclitaxel *n* = 31 (100%)	*P* value
Gender			0.212^*^
Male	19 (59%)	23 (74%)	
Female	13 (41%)	8 (26%)	
Age (years)			0.318^b^
Median	67 (32 ~ 74)	63 (31 ~ 77)	
< 60	20 (63%)	12 (39%)	
≥ 60	12 (37%)	19 (61%)	
Tumor location			0.535^*^
Gastro-esophageal junction	10 (31%)	12 (%)	
Gastric	22 (69%)	19 (%)	
Pathological types			1.000^a^
Adenocarcinoma	31 (97%)	31 (100%)	
Squamous cell carcinomas	1 (3%)	0 (0%)	
Clinical stage			0.514^*^
III	7 (22%)	9 (29%)	
IV	25 (78%)	22 (71%)	

^*^Chi-square test

^a^Fisher’s exact test

^b^Mann–Whitney *U* test

### Response Evaluation

Therapeutic response was observed in 32 cases in group A and 31 cases in group B. All the included cases were treated with at least 2 cycles of chemotherapy. A statistically significant difference in overall response was found between groups A and B*.* ORR was 31% and 10% in groups A and B, respectively (*P* = 0.034*)*.DCR was 91% and 81% in groups A and B, respectively (*P* = 0.440) (as shown in Table 2).

**Table 2:** The response rate of patients with advanced gastric cancer in two groups

Response	Number (%)		*P* value
	NPMP *n* = 32 (100%)	Paclitaxel *n* = 31 (100%)	
CR	1 (3)	0 (0)	…
PR	9 (28)	3 (10)	
SD	19 (59)	22 (71)	
PD	3 (9)	6 (19)	
ORR	10 (31)	3 (10)	0.034^*^
DCR	29 (91)	25 (81)	0.440^*^

*NPMP* nanoparticle polymeric micellar paclitaxel, *CR* complete response, *PR* partial response, *SD* stable disease, *PD* progressive disease, *ORR* objective response rate, *DCR* disease control rate

^*^Chi-square test

### Toxicity Assessment

There was no statistically significant difference in adverse reactions between the two groups (*P* > 0.05), a mild reduction in the incidence of adverse reactions in group A was found when compared to group B. The most common treatment-related adverse reactions at any level were anemia (41%) in group A versus (42%) in group B. Others include nausea and vomiting (22% versus 26%) and liver dysfunction (22% versus 39%) in group A and group B (as shown in Table 3).

**Table 3:** Toxicity evaluation in patients with advanced gastric cancer in two groups

Type of toxicity	NPMP (*n* = 32)					Paclitaxel (*n* = 31)					*P* value^*^
	I	II	III	IV	Total/(%)	I	II	III	IV	Total/(%)	
Hematological toxicity											
Leucopenia	3	0	1	0	13	4	1	0	0	16	0.700
Anemia	7	5	1	0	41	8	2	3	0	42	0.920
Thrombocytopenia	2	0	0	0	6	1	0	0	0	3	0.576
Non-hematological toxicity											
Nausea/vomiting	4	3	0	0	22	3	5	0	0	26	0.644
Diarrhea	2	0	0	0	6	2	1	0	0	10	0.597
Dyspnoea	0	0	0	0	0	0	1	0	0	3	0.310
Liver dysfunction	6	1	0	0	22	9	1	2	0	39	0.128
Renal function abnormality	1	0	0	0	3	0	0	0	0	0	0.325
Allergic reactions	1	0	0	0	3	2	1	0	0	10	0.283

^*^Mann–Whitney *U* test

## Discussion

Chemotherapy is the most effective treatment option for patients with AGC who are not suitable for surgery [17]. According to previous randomized studies, chemotherapy for AGC (especially recurrent or metastatic disease) is now widely accepted as a palliative treatment that prolongs survival and improves quality of life [18]. High response rates can be achieved with combined chemotherapy. In these regimens, 5-fluorouracil (5-FU)-based combinations yielded remission rates of 20% to 64% [7]. And fluoropyrimidine plus cisplatin is considered the primary recommended regimen for the first-line treatment of AGC [19]. However, this regimen is toxic and is not well tolerated by many patients with poor physical state, especially elderly patients. Instead, oxaliplatin was shown to be an effective agent for patients with gastric cancer, at least as effective as cisplatin, and is associated with slightly less toxicity and better tolerability [20]. In addition, several studies suggest that the modified FOLFOX-6 regimen could be effective in patients with AGC, with overall response rates ranging from 31.8 to 40.2%, median progression-free survival (PFS) ranging from 3.5 to 6.0 months, and median OS ranging from 9.2 to 13.0 months [21, 22]. Although oxaliplatin is better tolerated compared with other platinum compounds, it still has significant a toxic side effect, e.g., neurotoxicity.

Moreover, recent advances in the understanding of molecular genetics have led to the development of molecularly targeted therapies for gastric cancer, resulting in improved prognosis and quality of life for patients. Currently, trastuzumab remains the only such drug that is effective in combination with cytotoxic chemotherapy for the treatment of gastric cancer. The addition of trastuzumab to the treatment of human epidermal growth factor receptor 2 (HER2)-positive gastric cancer significantly improves survival in first-line therapy. Ramucirumab, an antibody targeting vascular endothelial growth factor receptor 2, significantly improves PFS and OS and has been approved for use in the second-line treatment of gastric cancer [23]. Phase III trials indicated that the anti-PD-1 inhibitor nivolumab prolongs overall survival in patients with AGC. Navulizumab is the first PD-1 inhibitor to demonstrate superior OS, PFS benefit, and an acceptable safety profile when combined with chemotherapy versus chemotherapy alone in patients with previously untreated advanced gastric cancer, gastroesophageal junction cancer, or esophageal adenocarcinoma. Nivolumab plus chemotherapy represents a new standard first-line treatment for these patients [24, 25].

Several studies confirmed the efficacy and safety of paclitaxel as a single agent in the treatment of AGC [26, 27]. Furthermore, paclitaxel is synergistic with other medications in the combination treatment of AGC. Paclitaxel plus ramucirumab demonstrated the highest objective response rate (ORR) of 28% reported in the second line setting compared with 16% in the paclitaxel alone group (*P* = 0.0001) in the RAINBOW trial [28]. Treatment with paclitaxel plus ramucirumab yielded the highest overall response rate reported to date for patients with previously treated advanced GC [29]. For all patients, second-line treatment of ramucirumab significantly improved OS. For patients willing to receive more treatment-related toxicity, ramucirumab plus weekly paclitaxel is a reasonable choice. Several phase II–III studies, as well as retrospective studies, have shown that paclitaxel-based regimens, including 5-FU and/or platinum compounds or etoposide, had high response rates of 32–65%, with a median survival of 6.8–14 months in patients with AGC [30–33]. And it was reported that infusion of high-dose 5-FU was an effective and well-tolerated treatment for patients with AGC and could be considered a basic component for this combination [34]. The non-overlapping toxicity profiles of paclitaxel and infused 5-FU, as well as the schedule-dependent synergistic effects between these agents against human GC cells, suggest a potential value of testing this combination in the treatment for patients with AGC [35]. In 2007, the combination of paclitaxel, 5-fluorouracil, and leucovorin was clinically investigated by Ninomiya et al. as first-line treatment for patients with AGC [36]. This phase II study with 65 patients reported an overall response rate of 38.6%, with 3.5% CR, 35.1% PR, and 43.9% SD. The median survival was 329 days, and a 1-year survival rate was 47.4%. Hematological and non-hematological toxicities were well tolerated. Other researchers studied the combination of paclitaxel with 5-FU and achieved objective response rates from 31 to 65%, with a median OS from 8.8 to 14 months [37–41]. Noticeably, paclitaxel in these researches was first-line administered, but doses or frequencies are variant: weekly, biweekly, or triweekly, and 5-FU was also variant from high-dose injection to continuous infusion, with or without leucovorin. On this background, Sakamoto et al. conducted a comprehensive review and suggested that depending on the schedule of paclitaxel administration, weekly injections appear to be less toxic and more effective than administered triweekly [42]. Regardless of the dosage or route of administration, regimens containing paclitaxel as well as 5-FU and leucovorin could be an effective first-line treatment for patients with AGC. However, these East Asian studies are too old, and there is no international consensus that paclitaxel, 5-fluorouracil, and leucovorin can be considered first-line therapy in patients with AGC.

Recently, how to reduce the allergic reaction caused by a paclitaxel-containing regimen has continuously been a hot issue. With the development of nanotechnology, a variety of nanocarrier platforms are established to improve the delivery efficiency of anticancer drugs [43, 44]. Polymeric micellar nanoparticle is one of the paclitaxel nanocarrier platform with excellent clinical performance [45]. And NPMP provides a solvent-free preparation for paclitaxel. Because tumor penetration ability is inversely proportional to nanoparticle size, polymeric micellar paclitaxel (about 20 nm) was reported to penetrate tumor cells easier and faster than nanoparticle albumin-bound paclitaxel (130 nm) [46]. Moreover, an evaluation of clinical trial data showed that NPMP improved ORR and progression-free survival (PFS) in patients with non-small cell lung cancer as a first-line treatment and did not show additional toxic effects after increasing the dosage of paclitaxel [15]. Possible mechanism is that NPMP could target tumor cells through enhanced permeability and retention effects and significantly reduce the retention of paclitaxel in the bloodstream, thereby improving drug uptake and accumulation in tumor tissue [14].

In our current study, when we focused on the clinical efficacy and toxicity of NPMP in combination with 5-fluorouracil and leucovorin in treatment for patients with AGC, ORR of NPMP was 31%, higher than 10% of conventional paclitaxel group with statistical significance. The incidence of anemia, leucopenia, nausea, vomiting, diarrhea, liver dysfunction, and allergy in the NPMP group was lower than those in the conventional paclitaxel group. Importantly, patients should take premedication before paclitaxel but do not need premedication before NPMP.

In conclusion, NPMP is superior to paclitaxel when combined with 5-fluorouracil and leucovorin in treating patients with advanced gastric cancer. NPMP with good anti-tumor activity and tolerable toxicities is a promising treatment option instead of conventional paclitaxel when patients with AGC need to be treated with paclitaxel-containing drugs. However, these are the results of a retrospective study and should be further investigated by prospectively designed randomized clinical trials with a larger sample size.

## Data Availability

Email can be sent to the following address to obtain shared data: 1808208607@qq.com
